# Development of a Single Thermal Cycling Multiplex RT-qPCR Assay for Rapid Arbovirus Detection

**DOI:** 10.3390/diagnostics16193205

**Published:** 2026-10-02

**Authors:** Róger J. Costa, Andreas Stocker, Cleyde Marconi, Gubio S. Campos, Silvia Sardi, Abevailton Miranda, Carlos Brites, Wei-Kung Wang, Eduardo M. Netto

**Affiliations:** 1 Programa de Pós-Graduação em Medicina e Saúde (PPgMS), Complexo Hospitalar Universitário Professor Edgard Santos, Federal University of Bahia, Salvador 40110-060, Brazil; rogerjcosta@hotmail.com (R.J.C.); inbox@andreas-stoecker.de (A.S.); marconi.cleyde@gmail.com (C.M.); abevailtonsilva@gmail.com (A.M.); wangwk@hawaii.edu (W.-K.W.); nettoeduardom@hotmail.com (E.M.N.); 2 Laboratório de Pesquisa em Infectologia (LAPI), Complexo Hospitalar Universitário Professor Edgard Santos, Salvador 40110-060, Brazil; 3 Virology Laboratory, Health Sciences Institute, Federal University of Bahia, Salvador 40110-100, Brazil; gubiosoares@gmail.com (G.S.C.); sissardi@yahoo.com.br (S.S.); 4 Fundação Bahiana de Infectologia, Salvador 40110-160, Brazil; 5 Department of Tropical Medicine, Medical Microbiology and Pharmacology, John A. Burns School of Medicine, University of Hawaii at Mãnoa, Honolulu, HI 96822, USA

**Keywords:** RT-qPCR, multiplex, arboviruses, molecular biology

## Abstract

**Background:** The co-circulation of multiple arboviruses with similar clinical presentations poses a significant challenge for public health surveillance. This study aimed to develop and clinically validate a novel multiplex RT-qPCR assay for the simultaneous detection of five arboviruses in three reactions. **Methods:** A diagnostic accuracy study was conducted in Salvador, Brazil. Evaluation was performed using 254 prospective serum samples and 34 archived positive samples. The primers and probes for the assay were designed *in silico*, all targeting highly conserved regions of Dengue (DENV), Zika (ZIKV), Chikungunya (CHIKV), Oropouche (OROV), and West Nile (WNV) viruses. The protocol was optimized in three reactions with a single thermal cycle, comprising a tetraplex reaction (DENV, CHIKV, ZIKV, and hRNAseP, an endogenous control) and two singleplex reactions (OROV and WNV). **Results:** In prospective analysis, the assay identified 45 DENV, 3 CHIKV, and 9 OROV-positive cases. Diagnostic accuracy for DENV and CHIKV was 96% (95% CI: 93.3–98.0%), successfully detecting four DENV cases missed by the reference standard. Furthermore, incorporating OROV enabled the identification of nine cases that conventional triplex protocols would have overlooked. **Conclusion:** This single-cycling multiplex RT-qPCR assay enhances epidemiological surveillance by expanding detection beyond traditional triplex targets to include OROV and WNV. This operational optimization reduces turnaround times and prevents the underdiagnosis of emerging arboviruses, serving as a robust diagnostic tool for endemic regions.

## 1. Introduction

The epidemiological outlook for arboviruses in Brazil in 2024 was marked by a high morbidity and mortality burden. Dengue was the arbovirus disease with the highest incidence, with 6,601,253 probable cases reported and 6264 confirmed deaths reported in Brazil in 2024. Chikungunya fever followed, with 265,545 probable cases and 243 deaths, while Zika virus infection had a recorded incidence of 5337 probable cases and one confirmed death according to the Arbovirus Monitoring Panel—Ministry of Health [1]. The expansion of Oropouche fever stands out, with the number of confirmed cases in Brazil growing from 833 in 2023 to 13,853 in 2024, according to the Oropouche Epidemiological Panel—Ministry of Health, positioning Oropouche as an emerging arbovirus of great relevance, with cases (including fatal) detected in non-endemic regions [2,3].

Dengue, Zika, Chikungunya, Oropouche and West Nile diseases present a similar acute clinical picture, with fever ranging from moderate to high, headache, muscle and/or joint pain, nausea and vomiting. The signs and symptoms are self-limiting in most cases, but there are cases of neurological outcomes such as meningitis and encephalitis, hemorrhage and the risk of the infection affecting pregnancy, causing microcephaly [4,5].

Diagnosis of arboviruses can be carried out using serological and molecular methods. Serological methods are less specific, are susceptible to cross-reactions and cannot detect coinfections in a single test. The molecular method, which is more sensitive and specific, detects viral RNA from a few copies and allows the inclusion of several targets in a single reaction [5].

Early detection of arboviruses is essential for surveillance and management of signs and symptoms. The objective of this study was to develop a panel Reverse Transcription Real-Time Polymerase Chain Reaction (RT-qPCR) for the detection of Dengue (1–4) (DENV), Zika (ZIKV), Chikungunya (CHIKV), West Nile (WNV), and Oropouche (OROV) viruses with endogenous control in three reactions using same thermal cycle.

## 2. Materials and Methods

### 2.1. Ethical Statement

This is a diagnostic accuracy study that was carried out at the Infectious Diseases Research Laboratory (LAPI) of the Edgard Santos University Hospital (HUPES) at the Federal University of Bahia in Salvador, Brazil. The study was submitted to and approved by the Ethics and Research Committee of the Climério de Oliveira Maternity Hospital (CAAE No. 76231523.6.0000.5543 and CAAE No. 25.336.819.3.0000.5543/4.682.940).

### 2.2. Design of Primers and Probes

All available sequences for DENV, ZIKV, CHIKV, OROV and WNV were obtained from GenBank (https://www.ncbi.nlm.nih.gov, accessed on 23 July 2023) and then aligned using Clustal Omega 64 bits software (EMBL-EBI, Hinxton, Cambridgeshire, UK) manually integrated with SeeView 5.0. The characteristics of the primers and probes were evaluated using the DNA oligonucleotide analyzer (IDT DNA). Specificity was verified using BLAST (https://blast.ncbi.nlm.nih.gov/Blast.cgi, accessed on 23 July 2023). Oligonucleotide evaluation and editing were performed using Bioedit software version 7.7.1 (Bioedit, Carlsbad, Califórnia, USA).

We performed an analysis *in silico* using IDT DNA’s Oligo Analyzer (https://www.idtdna.com/calc/analyzer) and ThermoFisher’s Multiple Primer Analyzer (https://www.thermofisher.com/br/en/home/brands/thermo-scientific/molecular-biology/molecular-biology-learning-center/molecular-biology-resource-library/thermo-scientific-web-tools/multiple-primer-analyzer.html, ThermoFisher, Waltham, MA, USA) to assess self-dimers and hetero-dimers in the MasterOligoMix for DENV, CHIKV, ZIKV, and hRNAseP, as well as in the singleplex reactions (OROV and WNV). The analysis data are included in the Supplementary Material.

### 2.3. Study Population

Between February and July 2024, we invited to participate in this study patients aged 5 years or older who were treated at an emergency care unit in Salvador (Bahia) with signs and symptoms such as fever, joint pain, headache, vomiting, and others, present within the first 5 days. According to the differential diagnosis protocol established by the Brazilian Ministry of Health and the diagnostic standard implemented by the reference laboratory in the state of Bahia, all samples from patients with clinically suspected arboviral infections were directly included in the RT-qPCR test if they were collected within 5 days of symptom onset [6].

### 2.4. Specimens and RNA Extraction

The study included prospective samples collected in emergency departments and archived samples with confirmed results for Dengue or Chikungunya.

Prospective serum was immediately stored at −20 °C after centrifugation, transferred to an ultrafreezer at −80 °C within 24 h, and kept there until the date of processing. Archived serum samples of RT-qPCR testing positive for DENV (*n* = 30) and CHIKV (*n* = 4) stored at −80 °C were kindly provided by the Institute of Health Sciences (ICS) of the Federal University of Bahia.

Prospective and archived specimens were extracted semi-automatically via the magnetic bead method, using the Loccus Extracta^®^—RNA and Viral DNA Kit (MVXA-PU16 FAST; Loccus, Cotia, SP, Brazil) with 100 μL of serum, following the manufacturer’s instructions.

We included an endogenous control to verify the quality of the extracted RNA. Genetic material can degrade due to inadequate storage or inefficient extraction. The hRNAseP developed by the CDC (Centers for Disease Control and Prevention) was incorporated into the tetraplex RT-qPCR [7], which was extracted along with any potential viral RNA, serving as a control throughout the process and serving as a validator for a non-detectable sample.

### 2.5. One-Step Real-Time RT-qPCR Reaction and Thermocycling Conditions

The protocol was designed to be performed in three reactions within a single thermal cycle: one reaction using tetraplex RT-qPCR (DENV, CHIKV, ZIKV, and hRNAseP), and another one for OROV and WNV, respectively.

Each tetraplex reaction has a total of 25 μL, consisting of 8 μL of RNA extracted from the serum, 12.5 μL of the reaction mix (dNTPs 0.4 mM; MgSO_4_ 6 mM; Buffer 2x; ThermoFisher, Waltham, MA, USA), 1 μL of BSA (1 ng/μL), 0.28 μL of RNAse-free H_2_O, 0.52 μL of MasterOligoMix (MOM), 1.7 μL of MgSO4 (50x), and 1 μL of Superscript III with Taq Enzyme Mix (25x; ThermoFisher, Waltham, MA, USA). The MOM is composed of a mixture of all the primers and probes from DENV, CHIKV, ZIKV and the endogenous control, with an initial concentration of 100 μM. The final concentration of each oligonucleotide is described in Table 1.

The one-step RT-qPCR singleplex for the detection of WNV was composed of 6 μL of RNA extracted from the serum, 12.5 μL of the reaction mix (dNTPs 0.4 mM; MgSO_4_ 6 mM; Buffer 2x; ThermoFisher, Waltham, MA, USA), 1 μL of BSA (1 ng/μL), 0.5 μL of each primer, 0.375 μL of the probe, and 1 μL of Superscript III with Taq Enzyme Mix (25x; ThermoFisher, Waltham, MA, USA).

The OROV one-step RT-qPCR singleplex was composed of 6 μL of RNA extracted from serum, 0.425 μL of RNAse-free H_2_O, 1 μL of BSA (1 ng/μL), 12.5 μL of the reaction mix (dNTPs 0.4 mM; MgSO_4_ 6 mM; Buffer 2x; ThermoFisher, Waltham, MA, USA), 0.5 μL of each primer and 0.375 μL of probe, and 1 μL of Superscript III with Taq Enzyme Mix (25x; ThermoFisher, Waltham, MA, USA). The thermal cycle used for the tetraplex and the singleplex for OROV and WNV was the same: 50 °C for 10 min, 55 °C for 10 min, 94 °C for 3 min and 50 cycles of 94 °C for 20 s and 56 °C for 40 s. The use of two reverse transcription temperatures (50 °C and 55 °C) was intended to maximize cDNA (complementary DNA) synthesis efficiency. The higher temperature facilitates the resolution of RNA secondary structures, improving reverse transcriptase processivity and enhancing assay sensitivity, particularly when structured viral RNA is present.

The fluorescence threshold was established during assay validation using standard curves generated from serial dilutions of the target template. The threshold was automatically determined by the instrument software based on the amplification profiles obtained across the dilution series and was subsequently fixed at the validated value for all subsequent runs. CT values were therefore determined using the same threshold throughout the study. We consider a sample to be detectable up to CT 40; the thresholds were adjusted manually on CFX 96 after the end of the analysis at 400, 350, 50, 250 and 350 for CHIKV, DENV, ZIKV, OROV and WNV, respectively. We used a template-free control (H_2_O DNase/RNase free). Positive controls used in the experiments are described in Section 2.8. All runs were performed on a Bio-Rad CFX-96 (Bio-Rad Laboratories, Hercules, CA, USA).

### 2.6. Viral Cultures

Viral cultures of DENV and WNV were provided by the Evandro Chagas Institute, Brazil, and cultures of CHIKV, ZIKV and OROV were provided by the Virology Laboratory at University Federal of Bahia (Brazil) (GenBank MK474461, KX101067 and MF155931, respectively). The viruses were obtained in C6/36 cell culture. The supernatants were aliquoted and stored at −80 °C until their use in all experiments [2,8].

C6/36 cells from *Aedes albopictus* (ATCC CRL-1660) were propagated in Leibovitz’s L15 medium (Gibco^®^, USA) supplemented with 5% Fetal Bovine Serum (FSB, Invitrogen, Brazil), 10% tryptose phosphate broth, non-essential amino acid solution (Gibco^®^, USA), 100 U/mL penicillin, and 100 μg/mL streptomycin antibiotic solution (Sigma-Aldrich, Saint Louis, MO, USA). Cell cultures were incubated at 28 °C. CO_2_ was not required.

### 2.7. Amplification Efficiency and Dynamic Range of the Assay

Viral RNA extracted from serum or viral culture was subjected to RT-qPCR for the specific target. The RT-qPCR product was purified using the PCR Purification Mini Kit (ThermoFisher, USA) and quantified using L-Quant (Loccus, Brazil). For each virus, a serial dilution curve was created based on purified dsDNA from 10^−1^ to 10^−14^, using only one measurement. Standard curves were generated with cycle threshold (CT) values on the X-axis and the logarithm of the number of dsDNA copies per 1 μL on the Y-axis. Graphs were created on Graphpad Prism 9 (GraphPad, USA). Crucially, these standard curves were generated solely to evaluate the amplification performance and operating characteristics of the PCR assay, rather than for absolute target quantification. Based on the results of the dilution series, a, b, and R^2^ were obtained through linear regression. The slope was calculated using formula 1/a, and the efficiency was calculated using formula E = (−1 + 10(−1/slope)) ⋅ 100. All calculations were performed in Microsoft Excel 365 (Microsoft, USA).

### 2.8. Reference Standard

Viral cultures for CHIKV, ZIKV, OROV, WNV, and DENV were used as positive controls to validate the technique developed in this study, and the results were confirmed by RT-qPCR for WNV [9], OROV [10], CHIKV [11], ZIKV [12] and DENV [13]. All procedures involving viral cultures were performed in a Biosafety Level 2 laboratory, following institutional biosafety procedures and applicable Brazilian regulations.

Prospective samples collected at the emergency care unit were also evaluated by the Bahia reference laboratory using the Molecular ZC D-Tipagem commercial kit (Bio-Manguinhos, Rio de Janeiro, RJ, Brazil) or the Biomol ZDC kit (IBMP, Curitiba, PR, Brazil), both of which have targets for Dengue 1–4, Chikungunya, Zika plus and endogenous or internal control. These commercially validated RT-qPCR assays were used as the reference standard for evaluation of the diagnostic performance of the assay developed in this study. Benchmarking the developed in-house multiplex RT-qPCR assay against commercial kits in the Brazilian market aligns its analytical validation with the country’s unique epidemiological, economic, and operational context. Because locally validated assays face the same mutational dynamics as circulating regional arbovirus lineages, this approach mitigates potential biases from the geographic primer/probe mismatching common in international kits designed from global or synthetic strains. Consequently, utilizing national comparators ensures higher accuracy and relevance when evaluating diagnostic performance within the specific regional epidemiological landscape.

The samples that tested positive for OROV using our RT-qPCR were subsequently tested by viral isolation; positive culture was confirmed by OROV RT-PCR using primers targeting the M gene as described previously [14]. PCR amplicon-based full-genome sequencing was performed using the previously described primer pairs [14] with Illumina DNA library preparation and MiSeq sequencing v3 600 cycles, followed by assembly using Genious Prime and phylogenetic tree analysis to determine genotype/lineage.

The standard reference technique used to confirm the archived samples was singleplex RT-qPCR for DENV [13] and CHIKV [11].

### 2.9. Statistical Analyses

The diagnostic performance measures were calculated using the reference method as the comparator. Sensitivity was defined as the proportion of true-positive samples correctly identified by the evaluated assay and specificity was defined as the proportion of true-negative samples correctly identified by the evaluated assay. The sensitivity, specificity and accuracy analysis with their respective 95% confidence intervals (CI 95%) were calculated using the Diagnostic Test Evaluation Calculator online tool (https://www.medcalc.org/en/calc/diagnostic_test.php, version 23.7.1; accessed 23 March 2026, MedCalc Software, Ostend, Belgium), using the values of true positive, true negative, false positive and false negative, and the prevalence calculated based on the positive individuals divided by the total participants of the study [15]. A true-positive result was defined as a sample testing positive by both the evaluated assay and the reference method. A true-negative result was defined as a sample testing negative by both methods. A false-positive result was defined as a sample testing positive by the evaluated assay but negative by the reference method, and a false-negative result was defined as a sample testing negative by the evaluated assay but positive by the reference method.

## 3. Results

The sequence (5′ to 3′) and final concentration of the DENV, CHIKV, ZIKV, OROV and WNV primers and probes are described in Table 1. The regions of primers in the viruses, amplicon size and primer positions in the reference genome are reported in the Supplementary Material.

An assessment of cross-reactivity was performed using serum or viral culture with DENV, CHIK, ZIKV, WNV, OROV, YFV (yellow fever virus), using tetraplex RT-qPCR for DENV, CHIKV, ZIKV, and hRNAseP, and singleplex RT-qPCR for OROV and WNV. No cross-reactivity was observed among the viruses evaluated.

The clinical characteristics of all patients included in the study and the subgroup of patients with detectable results for DENV, OROV and CHIKV are presented in Table 2. A detailed clinical description of confirmed cases of OROV was reported by Mirand et al. [16].

### 3.1. Standard Curves of One-Step Multiplex and Singleplex RT-qPCR Assay

**Figure 1:**
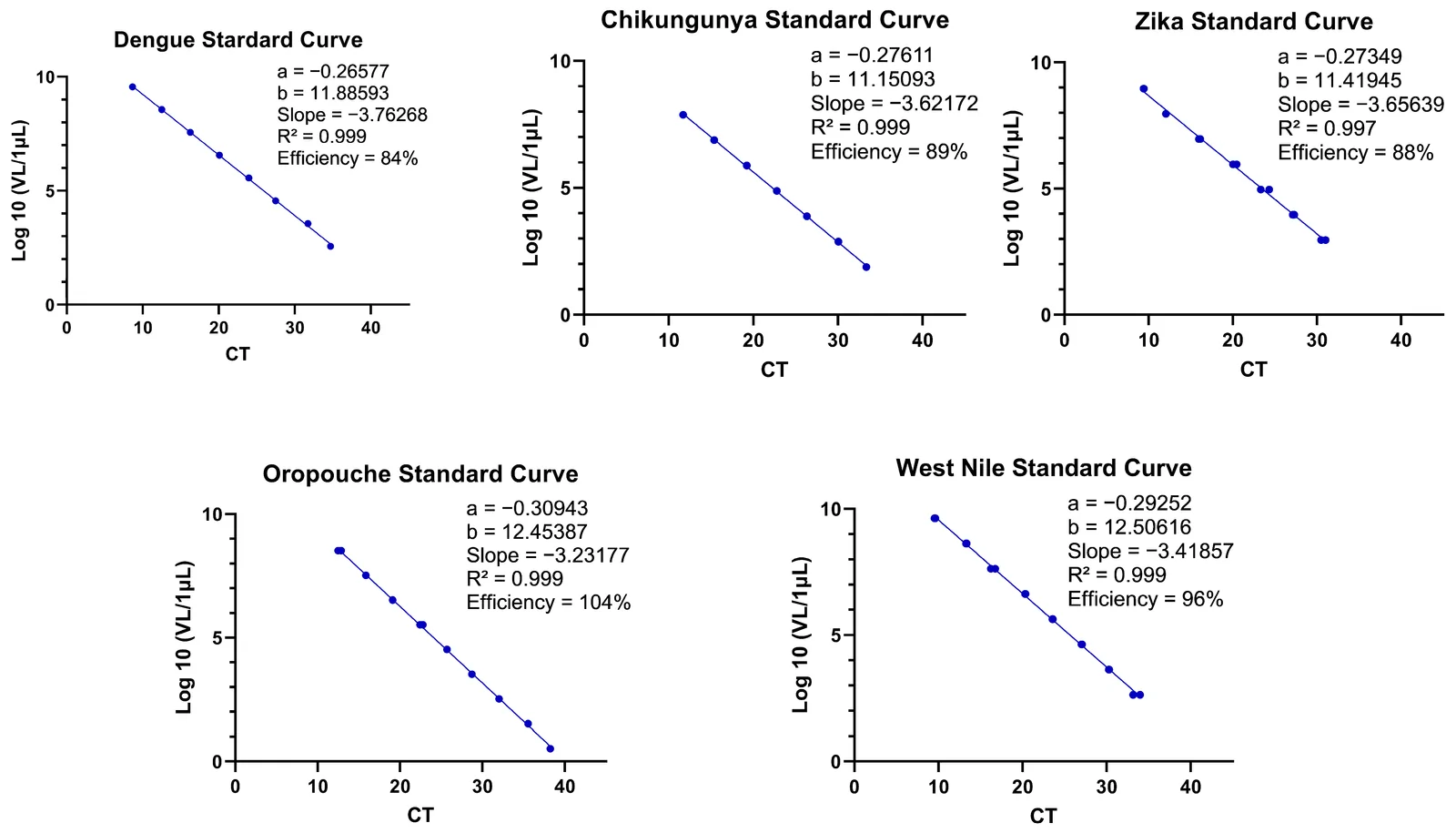
Characteristics of RT-qPCR assay. Ten-fold serially diluted DENV, CHIKV, ZIKV, OROV and WNV dsDNA standard. The figure shows the standard curves for DENV, CHIKV, ZIKV, OROV, and WNV, along with the efficiency metrics: R^2^, a, b, and slope. VL: viral load.

The standard curves were generated by plotting the cycle of quantification on the X-axis and Log 10 (viral load/1 µL) on the Y-axis. Figure 1 reports the linear regression, efficiency, determination coefficient (R^2^) and slope of standard curves evaluated on this study. The standard curves were generated to identify the operating characteristics of RT-qPCR, not for quantification.

The amplification efficiency of 104% for OROV was considered adequate for the intended purpose of the assay. Since the method was developed for the qualitative detection of targets, rather than for quantification, minor variations around the theoretical efficiency of 100% do not have a significant impact on the interpretation of the results.

Here, we report the lowest dilution point yielding detectable amplification in the serial dilution series for each virus. Copy numbers were estimated using the following formula: $Number\;of\;copies=\frac{DNA\;amount\;(ng)\;\cdot \;6.022\;\cdot \;10^{23}\;}{Molecular\;weight\;(g/mol)\;\cdot \;10^{9}}$, in which the molecular weight (g/mol) of each target and the dsDNA concentration measured by Qubit were used; next, the dilution factor corresponding to each virus was applied. Based on this approach, the lowest detectable values were 50 copies/μL for DENV, 10 copies/μL for CHIKV, 115 copies/μL for ZIKV, 5 copies/μL for OROV, and 50 copies/μL for WNV. These values represent the estimated copy numbers at the lowest dilution yielding detectable amplification under the experimental conditions evaluated and should not be interpreted as a formally established limit of detection.

### 3.2. Evaluation of Assay Using Clinical Samples

The diagnostic performance was evaluated using 254 prospective samples and 34 retrospective samples. In comparison with the reference method, the assay demonstrated a sensitivity of 90% (95% CI: 80–96%) for DENV (63/70) and 86% (95% CI: 42–100%) for CHIKV (6/7). Corresponding specificities for DENV and CHIKV were 97% (95% CI: 94–99%) and 100% (95% CI: 99–100%), respectively. Notably, among the 214 prospective samples initially classified as undetectable by the reference standard, our method identified seven additional positive cases (7 DENV).

Of the seven samples that tested positive using our method but negative using the reference method, three were confirmed by RT-qPCR after re-extraction, and one was confirmed based on a positive NS1 result. One sample was not retested due to a lack of material. The remaining two samples, which had CT values of 33.2 and 30.6 in the first round of RT-qPCR, were undetectable in the second retest. For the two samples that were positive in the reference test but negative in our test, a new RT-qPCR test was also performed using a new extraction, and the result remained undetectable.

All discordant samples in the analysis of archived samples had a valid hRNAseP value; the CT observed in the discordant samples ranged from 31.7 to 34.5, while most of the concordant samples had a CT below 30. We noted that the pre-analytical status of these samples differed from that of the prospectively analyzed samples, as they had been subjected to multiple cycles of freeze-thawing. It was not possible to perform a new analysis due to insufficient sample volume.

After resolving the disagreements, the updated measures for DENV were a sensitivity of 91% (95% CI: 81–96%) and a specificity of 99% (95% CI: 96–100%). Additionally, the overall accuracy of the technique, including both prospective and retrospective samples and the assessment for DENV and CHIKV, was 96% (95% CI: 93–98%).

**Figure 2:**
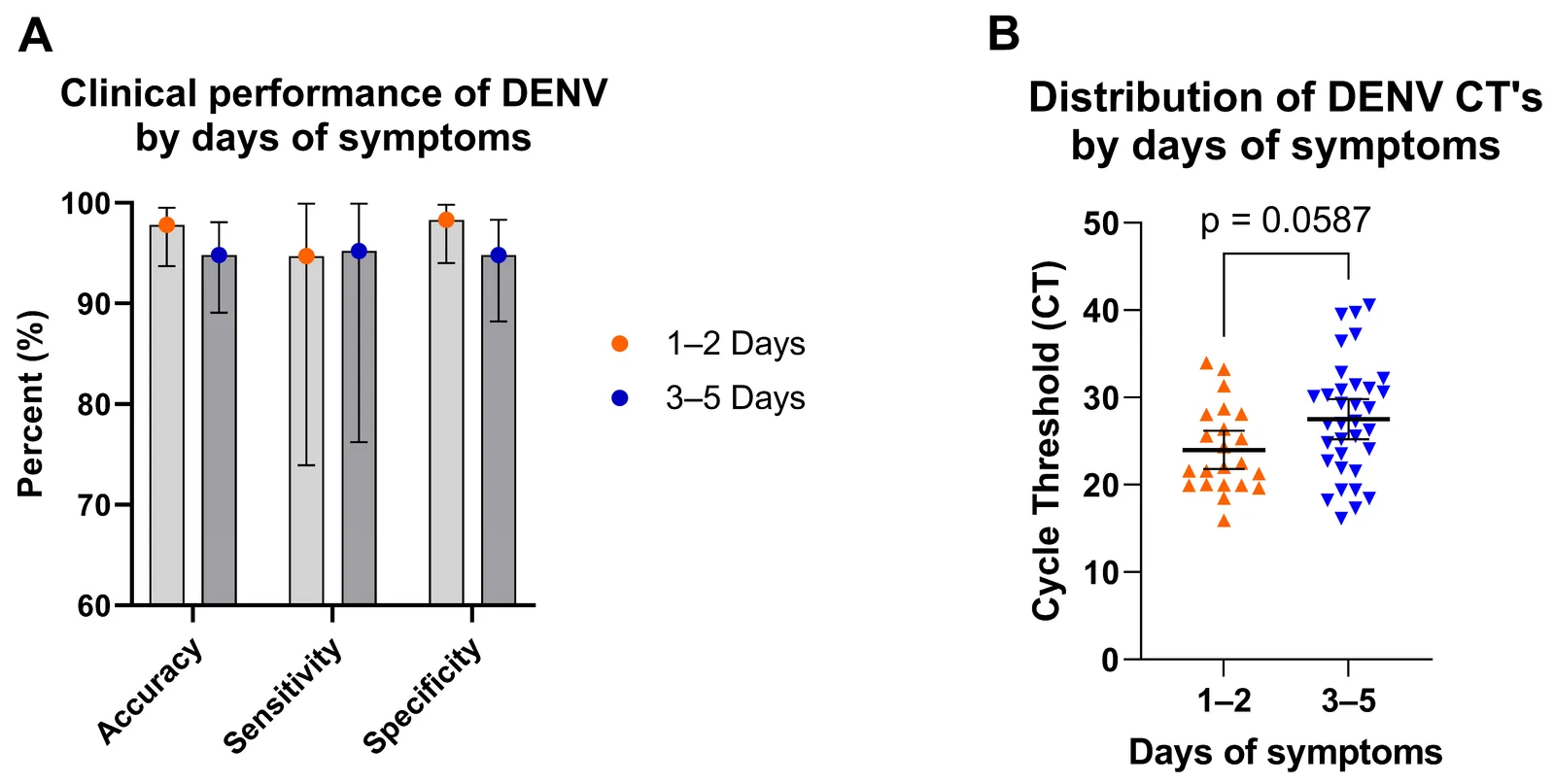
Clinical performance and Ct distribution of DENV RT-qPCR according to days of symptoms. Clinical performance of the DENV RT-qPCR assay stratified by symptom duration. (**A**) Accuracy, sensitivity, and specificity for samples collected within 1–2 days and 3–5 days after symptom onset. (**B**) Scatter plot showing Ct values by symptom duration. Orange represents Ct values between 1 and 2 days, and blue represents Ct values between 3 and 5 days of symptoms. The horizontal line represents the mean, and error bars represent 95% CI. The *p* value refers to the comparison between groups.

Figure 2 shows the clinical performance and distribution of CT values for DENV, respectively.

Of the nine samples detected for OROV by our RT-qPCR, six were confirmed by viral isolation, we also conducted a preliminary sequencing analysis showed they all belong to the recently emergent M1L2S2 lineage, consistent with the novel OROV reassortant of the 2022–2024 outbreak. The sensitivity was 100% (95% CI: 54–100%). The samples undetected by RT-qPCR had a median CT value of 34, which reduces the likelihood of successful isolation at higher CT values.

No ZIKV or WNV was detected in the prospective and retrospective samples analyzed, and no co-infections were observed among the viruses that tested positive. We conducted a clinical evaluation using serum samples with confirmed negative results, to which a positive viral culture was added. A 1:10 dilution of WNV from a CT 12 culture was performed up to a dilution of 10-7. For ZIKV, a 1:10 dilution of a CT 23 culture was performed up to a dilution of 10-3. All WNV (6/6) and ZIKV (3/3) dilution points, performed in duplicate, were detectable.

We compared the CT of DENV and CHIKV prospective and archived samples values using the Mann–Whitney test, which showed that there is no difference between the CT values of the samples tested using our technique and those of the reference method (*p* = 0.091). Furthermore, there is a moderate correlation (r = 0.417) between the CT values of the two techniques (*p* < 0.001; Spearman’s correlation test).

## 4. Discussion

This study describes the development and clinical validation of a protocol of multiplex RT-qPCR assay designed for the simultaneous detection of five arboviruses of high epidemiological relevance (DENV, CHIKV, ZIKV, OROV, and WNV) in three reactions. To our knowledge, this is the first RT-qPCR protocol for the detection of five arboviruses plus a control target in a single thermal cycle, representing an advancement over conventional protocols, in which the main multiplex RT-qPCR assays focus on the triple panel of DENV, ZIKV, and CHIKV [17,18,19]. The inclusion of OROV in the protocol proved particularly strategic for epidemiological surveillance, as our methodology was able to identify cases that would have been overlooked by traditional protocols. During the prospective analysis, a discrepancy was observed between the assay developed in this study and the reference method. Seven samples were initially identified exclusively by our methodology (mean CT = 33) and were not detected by the reference assay, after the disagreements were solved, and four cases were confirmed. Most of these patients were in the early acute phase (up to the third day after symptom onset), presenting classic clinical manifestations such as fever, myalgia, and headache, as well as symptoms such as retro-orbital pain, severe arthralgia, and asthenia.

In contrast, two samples were detected only by the reference technique, with CT of 27 and 36. These samples were collected on the second and fourth days of symptoms in patients presenting with fever, myalgia, headache, and cough.

The clinical evaluation of archived samples in our study demonstrated the high diagnostic sensitivity of the tetraplex RT-qPCR for detecting DENV and CHIKV. However, the analysis of archived specimens did not achieve 100% agreement. This result was also observed by Andrew et al. when using archived samples [20], a discrepancy we attribute to the detrimental effects of freeze–thaw cycles on RNA integrity. The reference RT-qPCR for these archived specimens yielded CT values exceeding 30, indicating low viral titters that are particularly susceptible to pre-analytical degradation during repeated handling. Despite these limitations, our protocol successfully identified most of these cases, reinforcing its analytical robustness.

Our technique did not serotype the detectable cases of Dengue; however, both the prospective and retrospective samples were serotyped by reference laboratories, which serotyped all cases as Dengue serotypes 1 and 2, consistent with the serotypes circulating in the region. Therefore, there is a need for further evaluation of the technique with serotypes 3 and 4. The assay was not validated using multiple CHIKV, WNV, and ZIKV strains, limiting the assessment of its performance across genetically diverse viral lineages. Further evaluation using a broader panel of strains is needed. Although the literature reports several multiplex protocols aimed at the simultaneous detection of DENV, ZIKV, and CHIKV [19,21,22,23,24,25,26,27,28,29], recent epidemiological trends highlight the urgent need to expand molecular surveillance to include emerging and re-emerging arboviruses, such as OROV, WNV, and Mayaro virus (MAYV). The implementation of an assay capable of identifying multiple viral targets in a single thermal cycle goes beyond technical optimization, establishing itself as a strategic tool in syndromic surveillance. In contrast to fragmented and sequential approaches [10,30,31], the methodology described here optimizes laboratory workflow, reduces turnaround time for results, and enables the detection of co-infections. The robustness of this protocol was corroborated by the identification of OROV cases that would have remained underdiagnosed under conventional protocols restricted to the classic triple panel.

Although individual assays and multiplex panels for WNV and OROV have been previously described [10,29,32,33], the objective of this study was not merely to introduce another diagnostic tool, but to provide an operationally optimized workflow. Performing reactions in separate tubes under a single, unified thermal profile prevents primer competition and the sensitivity trade-offs typically encountered in overcrowded single-well multiplex assays. Furthermore, this strategy overcomes the optical limitations of standard qPCR instruments, which are generally restricted to four or five simultaneous targets per well. By synchronizing all reactions within the same thermal program, our protocol enables the comprehensive screening of five arboviruses to be processed in a single run. This operational harmonization optimizes bench time, minimizes reagent waste, and accelerates turnaround times for critical epidemiological reports.

Compared with reference protocols used in Brazil (IBMP and Bio-Manguinhos [34]) and internationally (CDC) [19], the protocol developed in this study combines CHIKV, DENV, and ZIKV targets in a single reaction well, together with an endogenous control, whereas these reference protocols distribute these targets across two to four reaction wells. In addition, our protocol incorporates two further targets relevant to the current arbovirus landscape, which can be assessed within the same thermal cycle using two additional reaction wells. Thus, the proposed approach may increase testing comprehensiveness while reducing the number of thermal cycles required. However, its analytical and clinical performance has not yet been comprehensively validated, which limits direct comparison with established diagnostic protocols.

No positive cases of ZIKV and WNV were detected in our study, which is in agreement with the low prevalence of these viruses in Salvador, with only 25 laboratory-confirmed cases of ZIKV in 2024 and no cases of WNV reported between 2014 and 2024 [1,35].

### Strengths and Limitations

We evaluated comprehensive prospective clinical samples using a multiplex RT-qPCR protocol capable of detecting six targets in three reactions and single thermal cycle. The protocol can detect and distinguish five arboviruses in a single thermal cycle. Primer design was performed using a comprehensive multiple sequence alignment including all publicly available sequences for the target. This strategy enabled the identification of highly conserved regions, minimizing the impact of sequence polymorphisms on primer–template annealing. Consequently, the assay is expected to exhibit enhanced inclusivity and reduced false-negative rates across genetically diverse strains.

Our study is subject to limitations regarding its analytical and biological validation. First, a formal, absolute limit of detection (LoD) utilizing quantified RNA standards or viral stocks was not established. The current standard curves evaluate strictly the amplification efficiency and dynamic range using purified dsDNA, which does not account for RNA extraction or reverse transcription efficiencies. Although the analytical sensitivity was not evaluated using the gold standard, we have included the number of copies of lowest detectable dilution point obtained from the serial dilution series for each target. These values represent the lowest concentrations that yielded detectable amplification under the experimental conditions tested and should not be interpreted as a formal LoD. Future study should include a comprehensive LoD assessment using appropriate reference materials and replicate testing at low target concentrations to establish the assay’s detection limit according to accepted analytical validation standards. Second, regarding biological coverage, although the primers and probes were comprehensively designed *in silico* by aligning all available sequences in public databases to maximize conservation, experimental wet-lab validation was highly restricted by sample availability during the study period. Specifically, for the Dengue panel, only DENV-1 and DENV-2 were empirically tested, while the diagnostic performance against DENV-3, DENV-4, and distinct genetic lineages or strains of CHIKV, ZIKV, and WNV remains to be confirmed. Furthermore, due to the complete absence of natural infections in our cohort during the study period, the evaluation for ZIKV and WNV relied exclusively on contrived positive sera prepared from viral cultures. Consequently, further validation with authentic patient-derived clinical specimens is still strictly required to determine the real-world diagnostic parameters and clinical performance of the RT-qPCR assay for these two targets. Future investigations employing quantified RNA, a broader panel of reference viral strains, comprehensive reproducibility assessments, and robust clinical cohorts are necessary to fully validate and further characterize the performance of the protocol.

Our study detected only a few cases of CHIKV and OROV, and no cases of ZIKV or WNV; a clinical evaluation involving a larger number of samples is needed in the future to complete the validation of the panel. We conducted an in vitro cross-reactivity analysis limited to DENV, CHIKV, ZIKV, WNV, OROV, and YFV; a cross-reactivity analysis involving additional viruses will be necessary in the future.

## 5. Conclusions

In summary, this study describes the development and preliminary clinical evaluation of a novel, single-thermal-cycle multiplex RT-qPCR assay for the simultaneous detection of DENV, CHIKV, ZIKV, OROV, and WNV, alongside endogenous control in three reactions. While individual molecular assays for OROV and WNV are well-established, the primary operational advantage of this protocol lies in the harmonization of cycling conditions, allowing all targets to be screened simultaneously on a single PCR plate. By expanding the diagnostic scope beyond the conventional triplex panels (DENV, ZIKV, and CHIKV), our protocol demonstrates potential as a tool for epidemiological surveillance, successfully identifying OROV cases that would have otherwise remained undetected. Our results suggest that this multiplex protocol, which evaluates five arboviruses in a single run, is a promising alternative for the differential diagnosis of arboviruses. Its integration into routine laboratory surveillance could significantly improve the early detection and management of emerging outbreaks in endemic regions. Nonetheless, to fully establish the clinical utility of this assay, future studies must expand the validation cohort with a larger number of CHIKV and OROV samples, as well as incorporating WNV and ZIKV clinical specimens to complete the comprehensive validation of the panel.

## Figures and Tables

**Table 1:** Primers and probes used in the RT-qPCR assays.

ID	Sequence (5′ to 3′)	Final Concentration (μM)
Tetraplex—DENV, CHIKV, ZIKV and hRNAseP		
DENV-F1	CRG ATY TCT GAT GAA YAA CCA ACG	0.150
DENV-F2	CAG ATC TCT GGA AAA ATG AAC CAA CG	0.150
DENV-R1	GAG AAT CTC TTC RCC AAC TGT G	0.150
DENV-R2	GAG AAT CTC TTY GTC ARC TGT	0.150
DENV-R3	GAG AAT CTC TTC GTC AGC TGC	0.150
DENV-R4	GAG AAT CTC TTC ACC AAC CCY	0.150
DENV-P	FAM-CTW TCA ATA TGC TGA AAC GCG HGA GAA ACC G-BHQ	0.100
CHIKV-F	GAC GTG TTG TAC GTM GAC GA	0.150
CHIKV-R	GCA TTT TGC CTT CGT AAT GCA ACG	0.150
CHIKV-P	TexasRed-CCG AAR CAR TGC GGC TTC TTC AAT ATG ATG CAG ATG-BHQ	0.100
ZIKV-F	GAT TTG GAA ACG AGA GTT TCT GGT C	0.150
ZIKV-R	CAT GRC CCA GCA GRA GTC C	0.150
ZIKV-P	Cy5-CGG ATT GTC AAT ATG CTA AAA CGC GGA GTA GCC-BHQ	0.100
hRNAseP-F	AGA TTT GGA CCT GCG AGC G	0.100
hRNAseP-R	GAG CGG CTG TCT CCA CAA GT	0.100
hRNAseP-P	HEX-TTC TGA CCT GAA GGC TCT GCG CG–BHQ1	0.060
Singleplex OroV		
OROV-F01	GCA CAG AAG CTC TTA AGG TRC ATA G	0.200
OROV-F02	CAG AAG CCC TCA AAG TRC ACA G	0.200
OROV-R01	CCA GCT TTT MGA YTT ACA CAT CTG	0.200
OROV-R02	CCA GCT TTT AGA CTT RCA CAT YTG	0.200
OROV-P	TexasRed-GGT TAT AAR TCY CTA AGC AAA GC-MGB-NEQ	0.150
Singleplex WNV		
WNV-F01	GAG ART ATG GAG AGG TGA CAG TGG	0.200
WNV-F02	GTG ART ATG GTG AGG TYA CAG TTG	0.200
WNV-F03	GAG AAT ATG GAG AAG TRA CRG TGG	0.200
WNV-R01	CCA ACA GTC ATC ACR TAG TAT GC	0.200
WNV-R02	CCA ACT GAC ATA ACG TAR TAG GC	0.200
WNV-P	CY5-GTG ARC CAC GST CAG GGA TWG ACA C-BHQ3	0.150

Final concentration and sequence of the primers and probes for each target in the 5′ to 3′ direction.

**Table 2:** Clinical characteristics of prospective patients included.

Characteristics	Total *n* = 254	DENV *n* = 45	OROV *n* = 9	CHIKV *n* = 3
Age	31.5 (24.0–41.0	25.0 (21.0–33.0)	31.0 (25.0–46.0)	59.3 (9.5)
Sex, female	132 (52.0%)	22 (48.9%)	5 (55.6%)	0
Day of symptoms	2.0 (1.0–3.0)	3.0 (1.0–4.0)	2 (1.5–4.5)	1.33 (0.6)
Fever	228 (89.8%)	43 (95.6%)	9 (100%)	2 (66.7%)
Headache	217 (85.4%)	41 (91.1%)	9 (100%)	1 (33.3%)
Myalgia	215 (84.6%)	36 (80.0%)	8 (88.9%)	1 (33.3%)
Retroorbital pain	90 (35.4%)	21 (46.7%)	5 (55.6%)	1 (33.3%)
Diarrhea	89 (35.0%)	8 (17.8%)	4 (44.4%)	2 (66.7%)
Nausea	99 (39.0%)	15 (33.3%)	7 (77.8%)	0
Arthralgia	93 (33.6%)	13 (28.9%)	4 (44.4%)	2 (66.7%)
Vomit	43 (16.9%)	5 (11.1%)	0	0
Epigastralgia	59 (23.2%)	5 (11.1%)	1 (11.1%)	0
Cough	56 (22.0%)	4 (8.9%)	0	0
Asthenia	56 (22.0%)	9 (20.0%)	1 (11.1%)	1 (33.3%)
Odynophagia	48 (18.9%)	4 (8.9%)	0	0
Petechiae	32 (12.6%)	10 (22.2%)	4 (44.4%)	0
Low back pain	33 (13.0%)	7 (15.6%)	1 (11.1%)	1 (33.3%)
Exanthema	22 (8.7%)	4 (8.9%)	1 (11.1%)	0
Dizziness	22 (8.7%)	11 (24.4%)	0	0
Conjunctivitis	2 (0.8%)	0	0	0
Arthritis	2 (0.8%)	0	0	1 (33.3%)
Bleeding	5 (2.0%)	0	0	0

The table shows the clinical characteristics of all included patients, followed by a presentation of the positive cases for DENV, CHIKV and OROV.

## Data Availability

The data supporting the findings of this study are available within the article and its Supplementary Materials. Additional datasets generated and/or analyzed during the current study are available from the corresponding author upon reasonable request. The data are not publicly available due to privacy and ethical restrictions.

## Supplementary Materials

The following supporting information can be downloaded at: https://www.mdpi.com/article/10.3390/diagnostics16193205/s1. The 2×2 tables used to calculate accuracy, the primer design workflow, the assessment of primer and probe complementarity and the full sequences of the primers, as well as their locations and other characteristics, are available in “manuscript-supplementary”.

## Abbreviations

The following abbreviations are used in this manuscript:

RT-qPCR	Reverse Transcription Real-Time Polymerase Chain Reaction
CHIKV	Chikungunya virus
DENV	Dengue virus
OROV	Oropouche virus
ZIKV	Zika virus
WNV	West Nile virus
MAYV	Mayaro virus
BLAST	Basic Local Alignment Search Tool
MOM	MasterOligoMix
CI	Confidence interval
LoD	Limit of detection
cDNA	Complementary DNA
CT	Cycle threshold
VL	Viral load
