# Supplementary material for: Development of a Single Thermal Cycling Multiplex RT-qPCR Assay for Rapid Arbovirus Detection
Source: Diagnostics (Basel). 2026 Oct 2;16(19):3205. doi: 10.3390/diagnostics16193205 (PMC13664199; doi:10.3390/diagnostics16193205)
Supplement: Supplementary file 1 [file diagnostics-16-03205-s001.zip › Supp_Info_1.pdf]

1. Accuracy, sensitivity, and specificity of DENV testing between the 1st and 2nd days of symptoms

Test	Disease Present	n	Disease Absent	n	Total
Positive	True Positive	a= 18	False Positive	c= 2	a + c = **20**
Negative	False Negative	b= 1	True Negative	d= 116	b + d = **117**
Total		a + b = **19**		c + d = **118**	

Disease prevalence

If the ratio of cases in the Disease Present and Disease Absent groups does not reflect the disease prevalence, enter:

disease prevalence (%):

OK

Results

Statistic	Value	95% CI
Sensitivity	94.74%	73.97% to 99.87%
Specificity	98.31%	94.01% to 99.79%
Positive Likelihood Ratio	55.89	14.09 to 221.78
Negative Likelihood Ratio	0.05	0.01 to 0.36
Disease prevalence (*)	13.87%	8.56% to 20.81%
Positive Predictive Value (*)	90.00%	69.40% to 97.28%
Negative Predictive Value (*)	99.15%	94.51% to 99.87%
Accuracy (*)	97.81%	93.73% to 99.55%

(*) These values are dependent on disease prevalence.

2. Accuracy, sensitivity, and specificity of DENV testing between the 3rd and 5th days of symptoms

Test	Disease Present	n	Disease Absent	n	Total
Positive	True Positive	a= 20	False Positive	c= 5	a + c = **25**
Negative	False Negative	b= 1	True Negative	d= 91	b + d = **92**
Total		a + b = **21**		c + d = **96**	

Disease prevalence

If the ratio of cases in the Disease Present and Disease Absent groups does not reflect the disease prevalence, enter:

disease prevalence (%):

OK

Results

Statistic	Value	95% CI
Sensitivity	95.24%	76.18% to 99.88%
Specificity	94.79%	88.26% to 98.29%
Positive Likelihood Ratio	18.29	7.75 to 43.16
Negative Likelihood Ratio	0.05	0.01 to 0.34
Disease prevalence (*)	17.95%	11.47% to 26.12%
Positive Predictive Value (*)	80.00%	62.89% to 90.42%
Negative Predictive Value (*)	98.91%	93.07% to 99.84%
Accuracy (*)	94.87%	89.17% to 98.10%

(*) These values are dependent on disease prevalence.

3. Accuracy, sensitivity, and specificity of prospective and archived CHIKV samples

Test	Disease Present	n	Absent	n	Total
**Positive**	True Positive	a= 6	False Positive	c= 0	a + c = **6**
**Negative**	False Negative	b= 1	True Negative	d= 281	b + d = **282**
**Total**		a + b = **7**		c + d = **281**	

Disease prevalence

If the ratio of cases in the Disease Present and Disease Absent groups does not reflect the disease prevalence, enter:

disease prevalence (%):

OK

Results

Statistic	Value	95% CI
Sensitivity	85.71%	42.13% to 99.64%
Specificity	100.00%	98.70% to 100.00%
Positive Likelihood Ratio		
Negative Likelihood Ratio	0.14	0.02 to 0.88
Disease prevalence (*)	2.43%	0.98% to 4.94%
Positive Predictive Value (*)	100.00%	54.07% to 100.00%
Negative Predictive Value (*)	99.65%	97.86% to 99.94%
Accuracy (*)	99.65%	98.08% to 99.99%

(*) These values are dependent on disease prevalence.

4. Accuracy, sensitivity, and specificity of prospective and archived DENV samples before resolution of disagreements

Test	Disease Present	n	Absent	n	Total
**Positive**	True Positive	a= 63	False Positive	c= 7	a + c = **70**
**Negative**	False Negative	b= 7	True Negative	d= 211	b + d = **218**
**Total**		a + b = **70**		c + d = **218**	

Disease prevalence

If the ratio of cases in the Disease Present and Disease Absent groups does not reflect the disease prevalence, enter:

disease prevalence (%):

OK

Results

Statistic	Value	95% CI
Sensitivity	90.00%	80.48% to 95.88%
Specificity	96.79%	93.50% to 98.70%
Positive Likelihood Ratio	28.03	13.47 to 58.34
Negative Likelihood Ratio	0.10	0.05 to 0.21
Disease prevalence (*)	24.31%	19.47% to 29.68%
Positive Predictive Value (*)	90.00%	81.22% to 94.93%
Negative Predictive Value (*)	96.79%	93.72% to 98.38%
Accuracy (*)	95.14%	91.98% to 97.32%

5. Accuracy, sensitivity, and specificity of prospective and archived DENV samples after resolution of disagreements

	Disease				
**Test**	**Present**	**n**	**Absent**	**n**	**Total**
**Positive**	True Positive	a= 67	False Positive	c= 3	a + c = **70**
**Negative**	False Negative	b= 7	True Negative	d= 211	b + d = **218**
**Total**		a + b = **74**		c + d = **214**	

Disease prevalence

If the ratio of cases in the Disease Present and Disease Absent groups does not reflect the disease prevalence, enter:

disease prevalence (%):

OK

Results

Statistic	Value	95% CI
Sensitivity	90.54%	81.48% to 96.11%
Specificity	98.60%	95.96% to 99.71%
Positive Likelihood Ratio	64.59	20.95 to 199.15
Negative Likelihood Ratio	0.10	0.05 to 0.19
Disease prevalence (*)	25.69%	20.75% to 31.15%
Positive Predictive Value (*)	95.71%	87.87% to 98.57%
Negative Predictive Value (*)	96.79%	93.71% to 98.39%
Accuracy (*)	96.53%	93.71% to 98.32%

6. Accuracy, sensitivity, and specificity of prospective and archived DENV and CHIKV samples after resolving disagreements

	Disease				
**Test**	**Present**	**n**	**Absent**	**n**	**Total**
**Positive**	True Positive	a= 73	False Positive	c= 3	a + c = **76**
**Negative**	False Negative	b= 8	True Negative	d= 204	b + d = **212**
**Total**		a + b = **81**		c + d = **207**	

Disease prevalence

If the ratio of cases in the Disease Present and Disease Absent groups does not reflect the disease prevalence, enter:

disease prevalence (%):

OK

Results

Statistic	Value	95% CI
Sensitivity	90.12%	81.46% to 95.64%
Specificity	98.55%	95.82% to 99.70%
Positive Likelihood Ratio	62.19	20.17 to 191.68
Negative Likelihood Ratio	0.10	0.05 to 0.19
Disease prevalence (*)	28.12%	23.01% to 33.70%
Positive Predictive Value (*)	96.05%	88.76% to 98.68%
Negative Predictive Value (*)	96.23%	92.96% to 98.01%
Accuracy (*)	96.18%	93.27% to 98.08%

Criteria and primer design workflow

1. Oligo design for viral Qpcr

First, viral genomes that are as complete as possible—covering 80% to 90% of the genome length—are obtained from the GenBank repository using the ACNUC tool (Prabi — Pôle Rhône Alpes de Bioinformatique).

2. Alignment and Consensus Generation

The selected sequences are aligned using Clustal Omega 64-bit, manually replacing the 32-bit version of this software in Seaview 5.0. Next, two consensus sequences are generated in BioEdit 7.7.1: one that includes all bases and another with a 5% threshold. These alignments are not used directly for oligonucleotide design, but rather to identify candidate regions.

3. Selection of conserved regions

From the alignment, regions of interest are selected that are between 200 and 400 base pairs in length and exhibit sufficient conservation and base composition to support the implementation of a PCR reaction.

4. BLAST search and frequency analysis

Sequence fragments of up to 100 base pairs contained within these regions are used in BLAST searches to retrieve all available sequences that cover these loci. Using the NoteTab Light 7.2 editor, the frequency of each observed variant is determined, allowing us to assess whether a position is sufficiently conserved to serve as a primer or probe target.

5. Thermodynamic evaluation and specificity

Each proposed oligonucleotide undergoes melting temperature analysis using IDT's Oligo Analyzer. Subsequently, a positive search—restricted to sequences of the target virus—and a negative search—including all sequences retrieved by BLAST except the target sequence—are performed to verify specificity.

6. Final design criteria

The procedure ensures that, for each oligonucleotide, all available sequences in the region under consideration were incorporated into the design process, rather than relying solely on a subset of genomes. This ensures greater representativeness and robustness of the designed primers and probes.
